# Construction and Analysis of a Colorectal Cancer Prognostic Model Based on N6-Methyladenosine-Related lncRNAs

**DOI:** 10.3389/fcell.2021.698388

**Published:** 2021-08-19

**Authors:** Hanqian Zeng, Yiying Xu, Shiwen Xu, Linli Jin, Yanyan Shen, K. C. Rajan, Adheesh Bhandari, Erjie Xia

**Affiliations:** ^1^Department of Breast Surgery, The First Affiliated Hospital of Wenzhou Medical University, Wenzhou, China; ^2^Taizhou Hospital of Zhejiang Province Affiliated to Wenzhou Medical University, Linhai, China; ^3^Department of Breast Surgery, The Second Affiliated Hospital of Wenzhou Medical University, Wenzhou, China; ^4^Central Department of Zoology, Tribhuvan University, Kirtipur, Nepal

**Keywords:** N6-methylandenosine, colorectal cancer, long non-coding RNA, prognostic signature, prognostic model

## Abstract

Given the relatively poor understanding of the expression and functional effects of the N6-methyladenosine (m6A) RNA methylation on colorectal cancer (CRC), we attempted to measure its prognostic value and clinical significance. We comprehensively screened 37 m6A-related prognostic long non-coding RNAs (lncRNAs) with significant differences in expression based on 21 acknowledged regulators of m6A modification and data on 473 colorectal cancer tissues and 41 para-cancer tissues obtained from the TCGA database. Accordingly, we classified 473 CRC patients into two clusters by consensus clustering on the basis of significantly different survival outcomes. We also found a potential correlation between m6A-related prognostic lncRNAs and BRAF-KRAS expression, as well as immune cell infiltration. Then, we established a prognostic model by selecting 16 m6A-related prognostic lncRNAs via LASSO Cox analysis and grouped the CRC patients into low- and high-risk groups to calculate risk scores. Then, we performed stratified sampling to validate and confirm our model by categorising the 473 samples into a training group (*N* = 208) and a testing group (*N* = 205) in a 1:1 ratio. The survival curve showed a distinct clinical outcome in the low- and high-risk subgroups. We reconfirmed the reliability and independence of the prognostic model through various measures: risk curve, heat map and univariate and multivariate Cox analyses. To ensure that the outcomes were applicable to clinical settings, we performed stratified analyses on different clinical features, such as age, lymph node status and clinical stage. CRC patients with downregulated m6A-related gene expression, lower immune score, distant metastasis, lymph node metastasis or more advanced clinical staging had higher risk scores, indicating less-desirable outcomes. Moreover, we explored the immunology of colorectal cancer cells. The risk score showed positive correlations with eosinophils, M2 macrophages and neutrophils. In summary, our effort revealed the significance of m6A RNA methylation regulators in colorectal cancer, and the prognostic model we constructed may be used as an essential reference for predicting the outcome of CRC patients.

## Introduction

Colorectal cancer (CRC) is a common intestinal malignancy with the third highest morbidity (approximately 1.96 million cases) and third highest mortality (approximately 0.94 million cases) of all types of cancers worldwide (Sung et al., 2021). With improved survival and widespread screening for CRC in China, the increasing incidence trend is clear (Zhou et al., 2021). Due to high relapse and incomplete treatment, there is an urgent need to consider optimising new personalised targeted molecular treatments from a novel microscopic perspective.

RNA modification is an indispensable player in various processes of biological cellular activities. There are three main forms of nucleotide methylation: N6-methyladenosine (m6A), 5-methylcytosine [m(5)C] and pseudouridine. N6-methyladenosine (m6A) was identified as one of the most common and abundant RNA modifications after its discovery in the 1970s in eukaryotic messenger RNA (Adams and Cory, 1975) and viral nuclear RNA (Beemon and Keith, 1977). The methylation process is dynamically and reversibly performed by “writers” (demethylases) complexes and reversed by “erasers” (methyltransferases), and the effect of methylation depends on various “readers” (signal transducers), which mediate the effects of m6A on mRNA. Since 2019, the exploration of m6A RNA regulators in colorectal cancer reached a climax (Zhou et al., 2020). Gradually, the dual regulatory role of the m6A modification was discovered in a variety of tumours (He et al., 2018). The writers METTL3 and METTL14 were found to suppress proliferation and migration *via* the p38/ERK pathway (Deng et al., 2019). On the other hand, METTL3 facilitates tumour progression by stimulating long non-coding RNA (lncRNA) RP11 and Zeb1 expression (Li T. et al., 2019; Wu et al., 2019), maintaining SOX2 stability (Li T. et al., 2019) or maturation of pri-miR-1246 (Peng et al., 2019). Erasers mainly induce the progression and migration of CRC cells. FTO degrades miR-1266 or weakens the expression of STAT3, cyclin D, or MMPs to accelerate tumour growth (Shen et al., 2018). YTHDC2 and IGF2BP contribute to tumour metastasis by upregulating HIF-1α or c-Myc expression (Tanabe et al., 2016; Huang et al., 2018). YTHDF1 also hinders the Wnt/β-catenin pathway to increase tumorigenicity (Bai et al., 2019). Recently, a report showed that the reader LINRIS promotes tumour progression *via* the IGF2BP2-MYC axis and is regarded as a promising novel therapeutic target for CRC (Wang et al., 2019). Recently, many studies have demonstrated that lncRNAs modified by m6A have the capacity to function as oncogenic players or suppressors role of malignant tumours. However, how m6A regulators modify lncRNAs to affect tumour development is still unclear, and the mechanism of m6A modification of lncRNAs in colorectal cancer is particularly obscure. Consequently, determining the process and regulation of m6A-related lncRNA-dependent CRC will be valuable for determining a way forward toward effective targeted therapy.

In this study, we comprehensively collected clinical biological and genetic information from The Cancer Genome Atlas (TCGA) database, including 473 CRC specimens and 41 para-cancer tissues. Subsequently, 37 m6A-modified prognostic lncRNAs were confirmed using statistical methods. We also assessed the correlation between KRAS and BRAF gene expression and immune cell infiltration with prognosis by consensus clustering. With the goal of giving priority to clinical application, we established a prognostic model based on 16 genes filtered from 37 m6A-modified prognostic lncRNAs as a powerful and independent predictor of overall survival.

## Materials and Methods

### Datasets

We collected data from The Cancer Genome Atlas Colon Adenocarcinoma (TCGA-COAD) and Rectal Adenocarcinoma (TCGA-READ) cohorts, including the gene expression datasets (RNA-seq) and interrelated demographic (age and sex), clinicopathological (clinical M stage, pathologic T stage, pathologic N stage and infiltration of immune cells) and survival information. Patients with missing survival information were not included in the subsequent steps of the analysis.

### Bioinformatics Analysis

We downloaded thorough RNA-seq data from the TCGA-COAD and TCGA-READ cohorts, including 473 colorectal cancer tissues and 41 para-cancer tissues. To identify the target RNAs, we first selected hundreds of long non-coding RNAs relating to 21 acknowledged m6A-related genes, including expression data on writers (METTL3, METTL14, METTL16, WTAP, VIRMA [KIA1499], RBM15, RBM15B, and ZC3H13), erasers (FTO and ALKBH5) and readers (YTHDC1, YTHDC2, IGF2BP1, IGF2BP2, IGF2BP3, YTHDF1, YTHDF2, YTHDF3, HNRNPC, HNRNPA2B1, and RBMX). Then, we performed a Pearson’s correlation analysis to screen m6A-related lncRNAs (with | Pearson’s *R*| > 0.5 and *p* < 0.001) and implemented univariate Cox regression analysis to filter the prognostic m6-related lncRNAs in the dataset combining the survival information. Ultimately, we obtained 37 m6A-related prognostic lncRNAs and investigated the differential expression of genes (DEGs) between tumours and normal tissues. To explore the effect of m6A-related prognostic lncRNAs in the development of CRC, we categorised tumour tissues into two clusters according to 37 differentially expressed m6A-related prognostic lncRNAs by consensus clustering. We also performed a comparison to determine whether KRAS and BRAF, two crucial genes, were related to clinical characteristics. To better explore the distribution and function of immune cells, we compared the infiltration levels of 22 kinds of immune cells between the two clusters.

After least absolute shrinkage and selection operator (LASSO) Cox analysis on the 37 m6A-related lncRNAs in the TCGA cohorts, 16 filtered genes were identified to compose our prognostic model. We calculated the risk score for each patient according to the coefficient of each lncRNA. Therefore, all the patients were divided into high-risk and low-risk subgroups by the median value of the risk scores. Then, to increase the reliability and validity of our prognostic model, we randomly categorised the patients in the CRC database into a training group (*N* = 208) and a testing group (*N* = 205).

### Statistical Analysis

Kaplan–Meier survival curves and log-rank tests were performed to compare the survival status between various subgroups, comprising cluster 1 and cluster 2, different risk level subgroups in the testing and training groups and additional subgroups based on the clinical characteristics. Student’s *t*-test was utilised to compare KRAS and BRAF expression between clusters (cluster 1 and cluster 2) and tissues (normal and tumour), as well as risk scores (based on the 16 filtered m6A-related prognostic lncRNAs) between clusters (cluster 1 and cluster 2), immune score (high and low), distant metastasis (M0 and M1), lymph node metastasis (N0 and N1-3) and clinical stage (stages I–II and stages III–IV). Univariate and multivariate Cox regression analyses were used to evaluate the independence of our prognostic model regarding OS in the testing group and training group, respectively. The prognostic ability of our predictive model and other predictive factors (age, lymph node grade, and clinical stage) for OS were evaluated by receiver operating characteristic (ROC) curves and the area under the curve (AUC). The statistical analysis conducted in this study was performed using the R programming language (version 3.6.1) and SPSS Statistics 25 software. The related R codes were uploaded to the git-hub repository.

## Results

### Screening of m6A-Related lncRNAs

**FIGURE 1:**
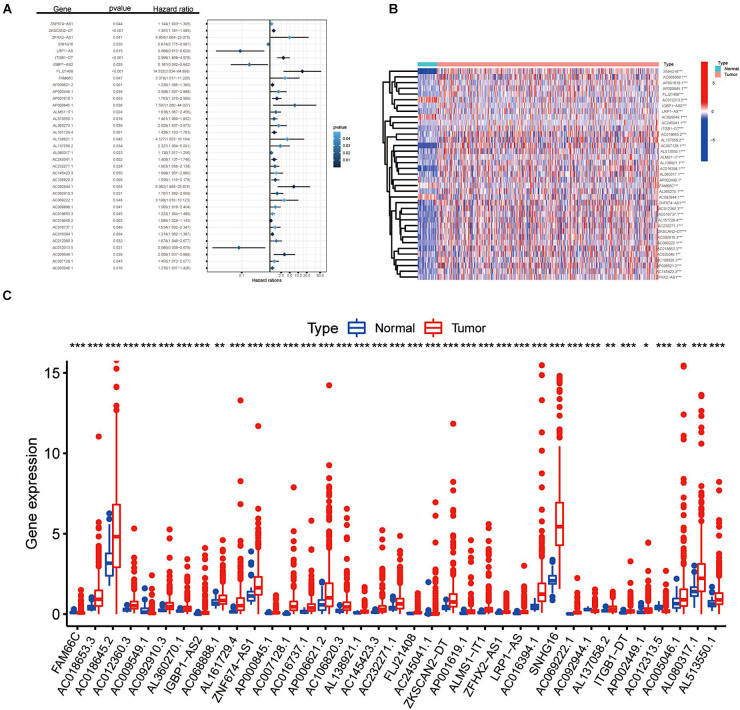
Screening of m6A-related lncRNAs. **(A)** Forest plot of the prognostic ability of the 37 filtered m6A-related lncRNAs included in the prognostic signature. **(B,C)** Heat map and the expression level of 37 m6A-related prognostic genes in CRC tumour tissues and normal tissues. *p < 0.05; **p < 0.01; and ***p < 0.001.

After a series of screening procedures, we obtained 37 m6A-related prognostic lncRNAs (Figure 1A). As illustrated in Figures 1B,C, the expression levels of m6A-modified lncRNAs in CRC and para-cancer tissues were obvious according to the analysis of DEGs. All 37 filtered lncRNAs were expressed differently with a significance of *p* < 0.05. There were 33 upregulated genes (SNHG16, AC068888.1, AP001619.1, AP000845.1, FLJ21408, IGBP1-AS2, LRP1-AS, AC245041.1, ITGB1-DT, AC018645.2, AC007128.1, AL137058.2, AL513550.1, ALMS1-IT1, AL138921.1, AC016394.1, AL080317.1, AP002449.1, AL360270.1, ZNF674-AS1, AC012360.3, AC016737.1, AL161729.4, AC232271.1, ZKSCAN2-DT, AC092910.3, AC069222.1, AC018653.3, AC005046.1, AC106820.3, AP006621.2, AC145423.3, and ZFHX2-AS1) and four downregulated genes (AC012313.5, AC009549.1, FAM66C, and AC092944.1), which shed light on the apparent variation of N6-methyladenosine modification in the development of colorectal cancer.

### The Type of Prognostic m6A-Related lncRNAs Varied by Prognosis and Clinical Characteristics

**FIGURE 2:**
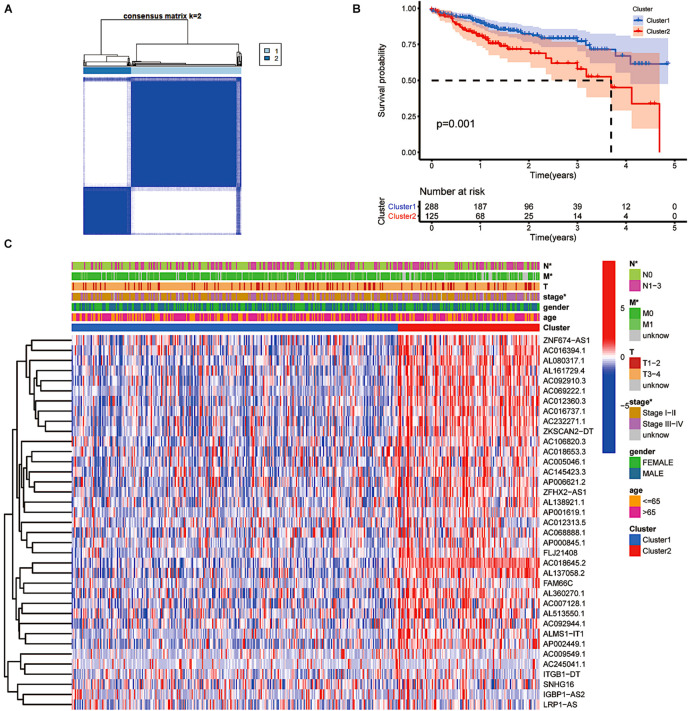
The type of prognostic m6A-related lncRNAs that vary in prognosis and clinical characteristics. **(A)** Consensus matrix for optimal *k* = 2. **(B)** Kaplan–Meier overall survival (OS) curves for patients in distinct clusters (*p* = 0.001). **(C)** Heat map of the associations between the expression levels of the 37 filtered m6A-related lncRNAs and clinicopathological features in the distinct clusters. *p < 0.05; **p < 0.01; and ***p < 0.001.

Through consensus clustering, we observed that the maximum AUC increment of CDF and the expression correlation of m6A-related prognostic lncRNAs were high within groups and low between groups. Thus, we determined that the *k* index was 2, namely, the number of clusters (Figure 2A). Cluster 1 (upregulation of m6A-related gene expression) scored higher, and cluster 2 (downregulation of m6A-related gene expression) scored lower. Kaplan–Meier survival curves showed that CRC patients in cluster 1 had longer overall survival (OS) (*p* = 0.001, Figure 2B). More than 50% of CRC patients in cluster 2 had a 3.8-year overall survival. To further investigate the relationship between clusters, clinical traits and expression levels of screened genes, we created a heat map suggesting that patients with higher scores in cluster 1 harboured significantly fewer lymphatic metastases, distant metastases and lower clinical tumour stages (*p* < 0.05, Figure 2C). CRC patients with a higher level of m6A-related lncRNA expression had fewer clinical features of tumour progression and better survival outcomes.

### The Differential Expression and Relevance of KRAS and BRAF Genes

**FIGURE 3:**
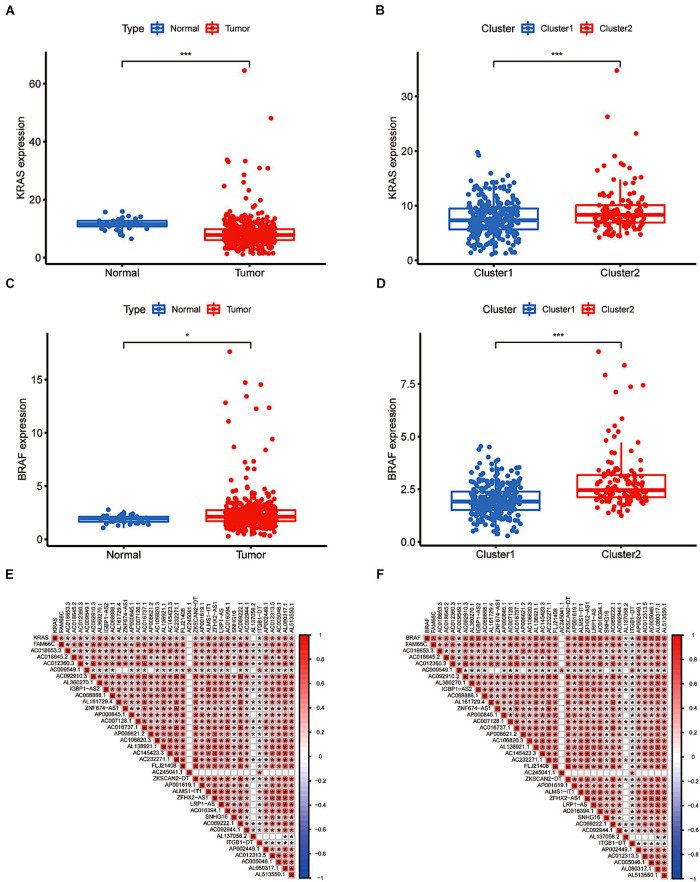
The differential expression and relevance of KRAS and BRAF genes. **(A)** KRAS downregulation in CRC. **(B)** The expression level of KRAS in cluster 1/2 subtypes. **(C)** BRAF upregulation in CRC. **(D)** The expression level of BRAF in cluster 1/2 subtypes. **(E,F)** The correlation of KRAS and BRAF with lncRNAs with m6A methylation. *P < 0.05; **P < 0.01; ***P < 0.001.

As key downstream sites of the EGFR signalling pathway, KRAS and BRAF gene mutations have been proven to play a pivotal role in the onset and development of CRC (Taieb et al., 2017; Dekker et al., 2019). The frequency and prognostic value of KRAS and BRAF mutations have piqued general public interest. The results indicated that KRAS expression was significantly lower in tumour tissue than in tissue obtained from normal subjects, providing evidence that KRAS gene mutation is an independent risk factor for the prognosis of colorectal cancer patients. The BRAF gene showed the opposite pattern (Figures 3A,C). Nevertheless, according to a subgroup analysis, we found that both BRAF and KRAS genes were consistently more highly expressed in cluster 2 (*p* < 0.001, Figures 3B,D). Both BRAF and KRAS were expressed at significantly lower levels in individuals with downregulated m6A-related lncRNAs than in those with upregulated m6A-related lncRNAs. In addition, we performed a correlation analysis between m6A-related prognostic lncRNAs and these two genes. As shown in Figures 3E,F, all the screened m6A-lncRNAs positively correlated with BRAF and KRAS genes except for AC009549.1, AC245041.1, AL137058.2, and ITGB1-DT, which showed a poor correlation with these genes. We hypothesised that the upregulation of m6A-modified lncRNAs related to prognosis might have a hidden connection with the KRAS-BRAF-EGFR signalling pathway and induce tumour cell angiogenesis, cell proliferation, invasion and metastasis, enriching the targets for the treatment of CRC (Martinelli et al., 2020).

### Immunological Analysis Between Distinct Clusters

**FIGURE 4:**
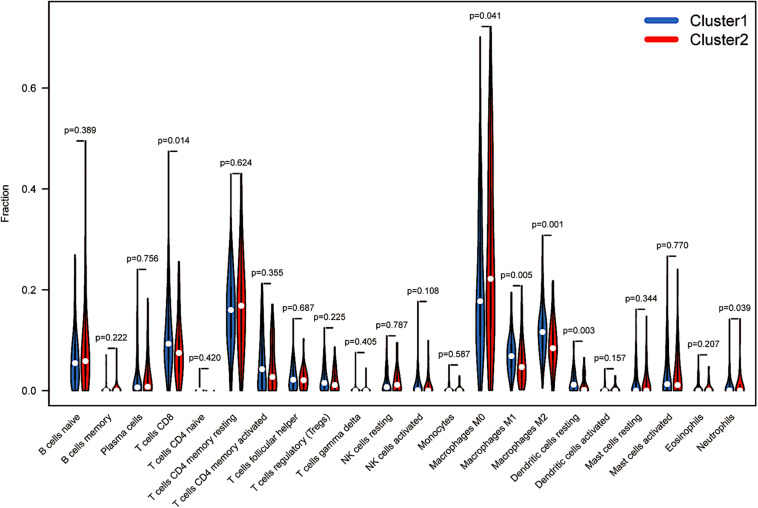
Immunological analysis between distinct clusters. The infiltration levels of 22 immune cell types in the cluster 1/2 subtype.

More recently, the immune status of the tumour microenvironment (TME) has become a topic of intense discussion. The proportion of 22 immune cell types also differed between the two clusters. Resting memory CD4 T cells and M0 macrophages accounted for more memory CD4 T cells than other cells. Cluster 1 showed higher levels of infiltration of CD8+ T cells, macrophages (M1 and M2), resting dendritic cells and neutrophils (*p* < 0.05, Figure 4). However, M0 macrophages in cluster 1 comprised a lower infiltrating fraction than those in cluster 2. Therefore, we speculated that the expression of m6A-modified lncRNAs might promote M0 macrophage active differentiation into M1 or M2 macrophages to counteract tumour cell activity and improve survival outcomes.

### The Construction of the Prognostic Model

**FIGURE 5:**
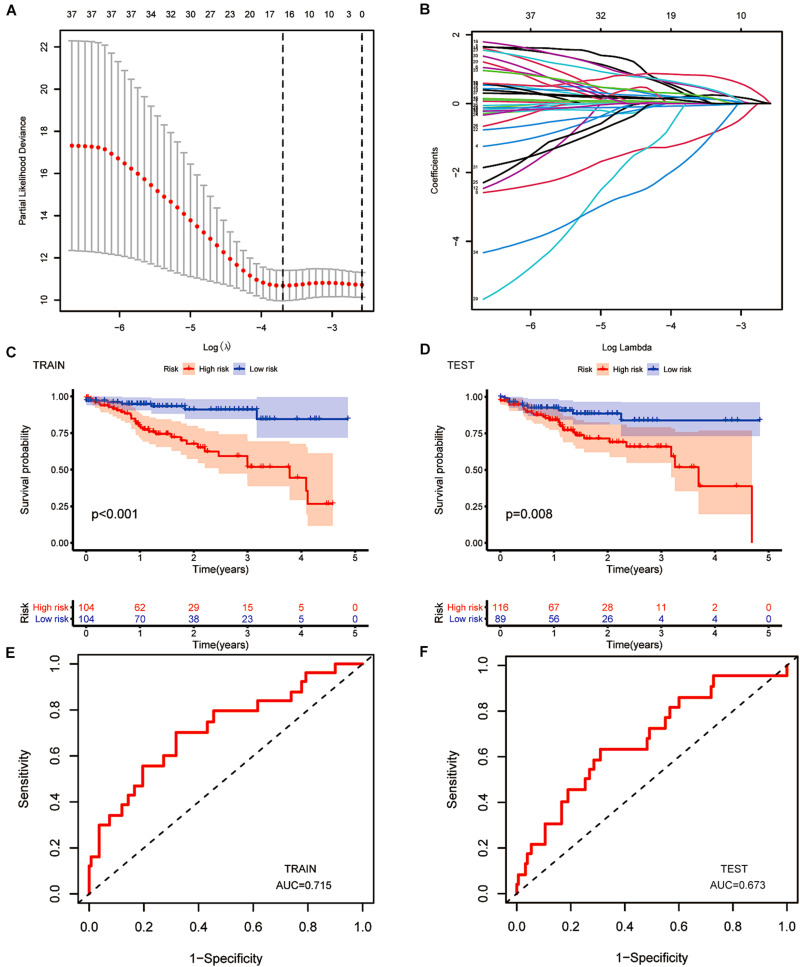
The construction of a prognostic model. **(A,B)** Least absolute shrinkage and selection operator (LASSO) regression was performed, calculating the minimum criteria. **(C,D)** Kaplan–Meier curves showed the same result: the high-risk subgroup had worse overall survival than the low-risk subgroup in the testing group (*p* < 0.001) and the training group (*p* = 0.008). **(E,F)** ROC curves of 16 m6A-related prognostic lncRNAs for predicting overall survival in the training group (AUC = 0.715) and the testing group (AUC = 0.573).

**FIGURE 6:**
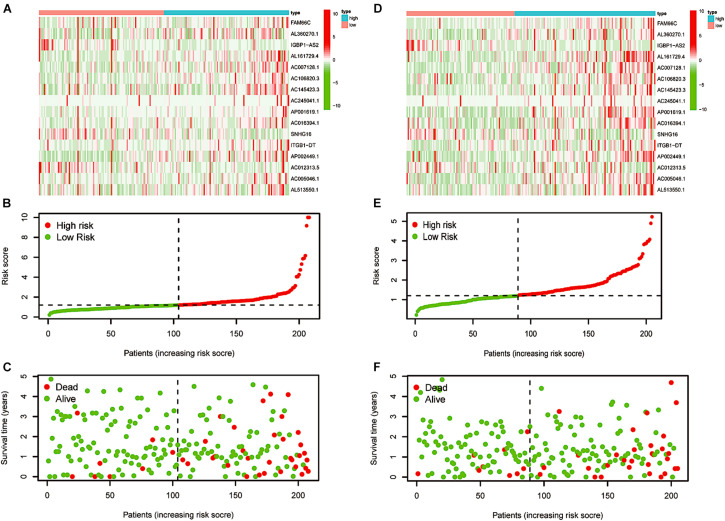
Re-verification of the prognostic model. **(A)** Distributions of risk scores of CRC patients in the training group. **(B,C)** Heatmap of the relationship between lncRNAs composed of the prognostic model and risk scores and that between lncRNAs composed of the prognostic model and survival status in the training group. **(D)** Distributions of the risk scores of CRC patients in the testing group. **(E,F)** Heatmap of the relationship between lncRNAs composed of the prognostic model and risk scores and that between lncRNAs composed of the prognostic model and survival status in the testing group.

After LASSO Cox analysis on the 37 m6A-related lncRNAs in the TCGA cohorts, 16 filtered genes were identified to compose our prognostic model (Figure 5A). They were FAM66C, AL360270.1, IGBP1-AS2, AL161729.4, AC007128.1, AC106820.3, AC145423.3, AC245041.1, AP001619.1, AC016394.1, SNHG16, ITGB1-DT, AP002449.1, AC012313.5, AC005046.1, and AL513550.1. The coefficients were 0.293783990289914, 0.20674487093833, −1.07314848883761, 0.150291468524777, 0.213403369658082, 0.177374784091726, 0.509171377590994, 0.0256044712477644, 0.540042712898157, 0.0148215168852506, −0.0593895376145353, 0.832117386651483, 0.257368853415027, −1.499057457211, 0.00475815225681812, and 0.0680978436662801. To increase the reliability and validity of our prognostic model, we randomly allocated the patients in the CRC database into a training group (*N* = 208) and a testing group (*N* = 205). We then calculated the risk score for each patient according to the coefficient of each lncRNA (Figure 5B). Therefore, all the patients were divided into high-risk and low-risk subgroups by the median value of risk scores. Kaplan–Meier survival curves revealed that CRC patients with higher risk scores had remarkably worse clinical outcomes, which was consistently observed in the training group (*p* < 0.001) and testing group (*p* = 0.008, Figures 5C,D). The ROC curves indicated that our prognostic model was a powerful and stable predictive tool for CRC patient survival (AUC = 0.715 for the training group, and AUC = 0.673 for the testing group; Figures 5E,F). The heat map in Figure 6A shows the general tendency of higher levels of m6A-related lncRNA expression in the high-risk group. The risk curve and the heat map indicate that the proportion of deaths in the high-risk group was far greater than that in the low-risk group (Figures 6B,C). This conclusion was validated in the testing group, as shown in Figures 6D–F, which is powerful proof of our prognostic model. These results prove that the proposed model is a good indicator of differentiation, is unbiased and is a source of reference.

### Factor Analysis of the Prognostic Model

**FIGURE 7:**
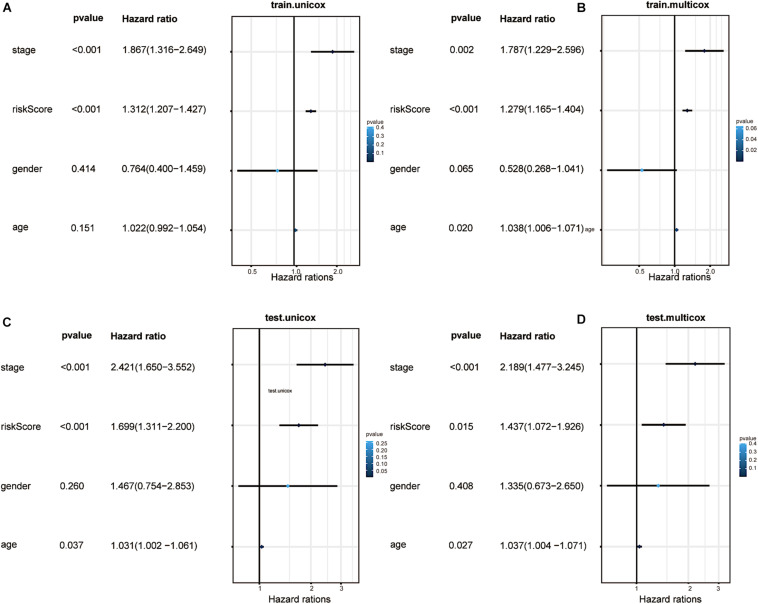
Factor analysis and confirmation of independence. **(A)** Univariate analyses illustrated that the risk score (based on the 16 m6A-related prognostic lncRNAs) was an independent prognostic predictor in the training group. **(B)** Multivariate analyses illustrated that the risk score was an independent prognostic predictor in the training group. **(C)** Univariate analyses illustrated that the risk score was an independent prognostic predictor in the testing group. **(D)** Multivariate analyses illustrated that the risk score was an independent prognostic predictor in the testing group.

To determine the independence of our predictive model in the prognostic analysis of CRC patients, we utilised univariate and multivariate Cox analyses. In the training and testing groups, the clinical stage and the risk score were the only two significantly relevant independent factors for OS (*p* < 0.05, Figures 7A–D). In addition, the factor of age analysed in the testing group seemed to be another independent prognostic element (*p* = 0.027). Sex seemed to be irrelative in both groups. Indeed, the model we built can independently predict the clinical prognosis of CRC.

### Confirming the Independence of the Prognostic Model

**FIGURE 8:**
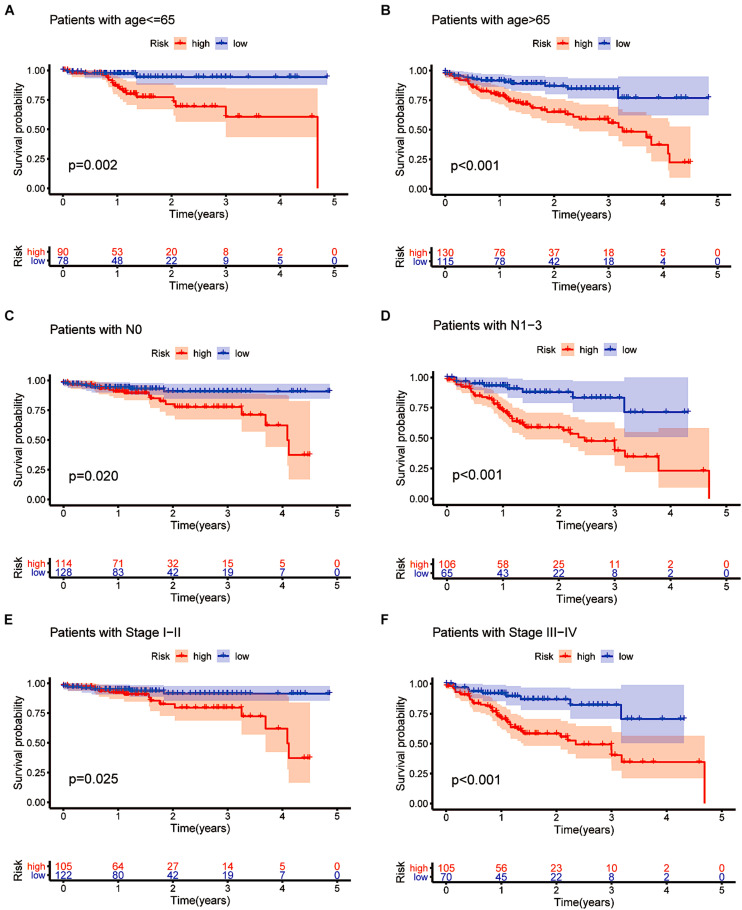
Confirming the independence of the prognostic model. **(A–F)** The model we constructed based on the 16 m6A-related prognostic lncRNAs retained its prognostic value in multiple subgroups of CRC patients (including patients aged ≤ 65 or >65 years, patients with N0 or N1-3 and patients with stages I–II or stages III–IV disease).

We also performed a stratified analysis on age, lymph node status and clinical stage to confirm whether the prognostic model retains the power to predict survival. CRC patients at higher risk showed a worse OS in the age ≤ 65 and age > 65 subgroups than patients at lower risk (*p* = 0.002 and *p* < 0.001, Figures 8A,B). Similarly, we evaluated the prognostic ability of N0 and N1-3. Patients without lymph node metastasis had a better prognosis (*p* = 0.020 in the N0 subgroup and *p* < 0.001 in the N1-3 subgroup, Figures 8C,D). As shown in Figures 8E,F, different outcomes between the stage I–II subgroup and the stage III–IV subgroup were obtained (*p* = 0.025 in the stage I–II subgroup and *p* < 0.001 in the stage III–IV subgroup). The subgroup analysis revealed that in the age subgroup, lymph node subgroup and clinical staging subgroup, the high-risk group was significantly different from the low-risk group. This is additional proof that our prognostic model may be regarded as a promising and independent tool to predict the clinical prognosis of CRC patients.

### The Relationship Between Risk Scores and Clinical Features

**FIGURE 9:**
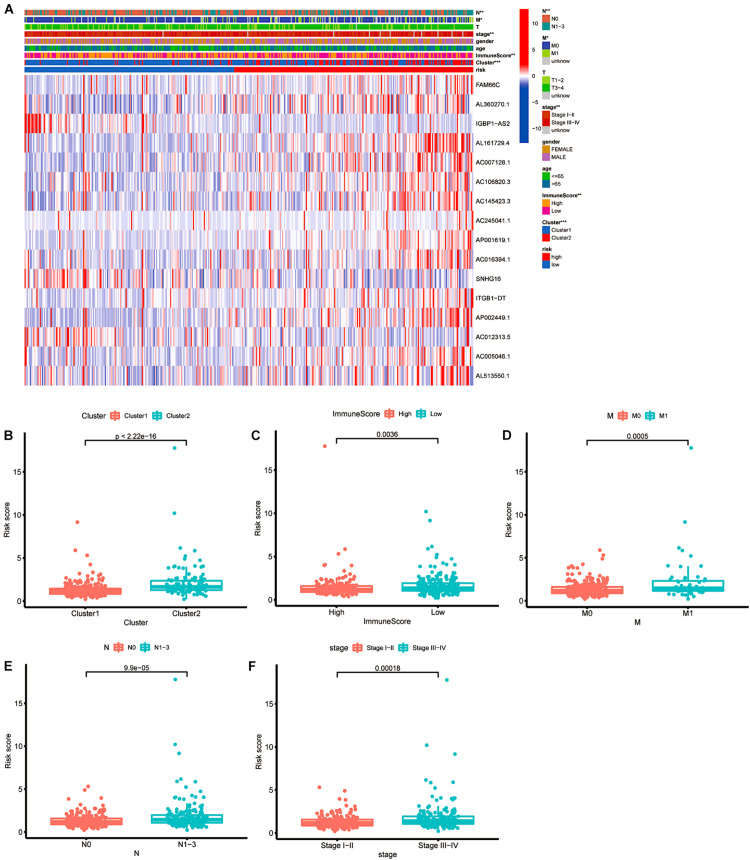
The relationship between risk score and clinical features. **(A)** Heatmap of the associations between the expression levels of the 16 m6A-related lncRNAs and clusters and clinicopathological features. *p < 0.05; **p < 0.01; and ***p < 0.001. **(B–F)** Patients with different clusters and clinicopathological features (including immune score, distant metastasis staging, lymph node metastasis staging and clinical staging) had different levels of risk scores, calculated based on the 16 m6A-related prognostic lncRNAs.

To apply the prognostic model to the clinical features, we performed a more specific analysis to determine whether the risk score has a relationship with clinicopathological information (Figure 9A). CRC patients in cluster 2 had a higher risk score, with a significant difference of *p* < 0.001 (Figure 9B). The box plot shown in Figure 9C indicates that different immune scores were also related to various risk scores (*p* = 0.036). Then, we compared the M stages, N stages and clinical stages. As shown in Figures 9D–F, there was a significant difference in risk scores (*p* = 0.005 in the M staging subgroup, *p* < 0.01 in the N staging subgroup, and *p* = 0.00018 in the clinical staging subgroup). CRC patients in cluster 2 with a lower immune score, distant metastasis, lymph node metastasis or more advanced clinical staging had higher risk scores, indicating less-desirable outcomes.

### Relationships Between the Risk Scores and Infiltration Abundances of Three Immune Cell Types

**FIGURE 10:**
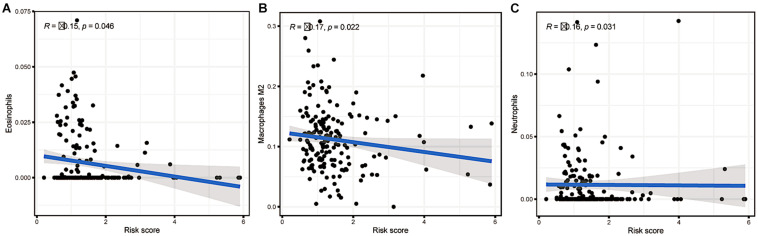
Relationships between the risk score and infiltration abundances of three immune cell types. **(A)** Eosinophils (*p* = 0.046), **(B)** M2 macrophages (*p* = 0.022), and **(C)** neutrophils (*p* = 0.031).

Then, we explored the immune microenvironment of colorectal carcinoma by elucidating three crucial types of immune cell infiltration in different risk classes. As the risk score increased, the levels of eosinophils, M2 macrophages and neutrophils in the body decreased significantly in the low risk group (Figure 10). Combining with prior analysis of immune cell infiltration between clusters, the overexpression of m6A-related lncRNAs presumably reduced the risk score by increasing the fraction of M2 macrophages. However, more investigation is needed to confirm this result.

## Discussion

Colorectal cancer is highly prevalent, and predicting the outcome is important. In recent years, increasing evidence has shown the significance of lncRNAs in tumourigenesis and cancer progression. Moreover, lncRNAs are also considered potential diagnostic and prognostic markers in cancer.

After witnessing the success of targeted molecular therapy in some tumour clinical applications, there has been increasing enthusiasm for research on the impact of lncRNAs on CRC. We identified 37 m6A-related prognostic lncRNAs from 473 CRC patients and added 16 of these RNAs to our prognostic model. Numerous studies have confirmed that SNHG16 acts as a tumour suppressor during CRC development by regulating tumour cell invasiveness and metastasis *in vivo* and *in vitro* (Qi et al., 2015; Li Y. et al., 2019). Further studies have shown that SNHG16 is regulated by Wnt transcription factors (Christensen et al., 2016). SNHG16 expression promotes the epithelial-mesenchymal transition through the miR-124-3p/MCP-1 (Ghasemi et al., 2020), miR-302a-3p/AKT axis (Ke et al., 2020) or miR-132-3p/USP22 pathway (He et al., 2020) to enhance invasiveness and resistance. It has been proven that the lncRNA FAM66C activates the EGFR-ERK pathway to promote cell proliferation in prostate cancer (Xie et al., 2020). In addition to tumour growth, drug resistance is an important consideration in the clinic, and Zhang et al. (2021) constructed ceRNA networks including FAM66C, which is associated with tumour mutation burden (TMB), for predicting drug resistance in melanoma. In a study of oesophageal cancer, AC007128.1 was regarded as a prognosis-related lncRNA (Liu et al., 2020). AP001619.1 is involved in the ceRNA network related to overall survival in colon cancer (Huang and Pan, 2019). A recent study revealed ITGB1-DT as an oncogenic lncRNA in lung adenocarcinoma (LUAD) that activates the ITGB1-DT/ITGB1/Wnt/β-catenin/MYC positive feedback loop (Chang et al., 2021). Several genes have been reported to play a role in cancer progression, but articles regarding colorectal cancer remain rare. The specific influencing mechanism of m6A modification on prognostic lncRNAs is unclear. Furthermore, the overall mechanism of lncRNAs in colorectal malignancy is not yet completely understood. The polygenic model is a preferred way to comprehensively clarify the relationship.

For this study, we enrolled 473 CRC patients to estimate the prognostic value of m6A-related lncRNAs. Performing DEG analysis, we screened 37 lncRNAs with the ability to predict survival outcomes. Through the foundation of consensus clustering, we determined the prognostic heterogeneity among clinical characteristics and immune cell infiltration levels. Sixteen of the 37 lncRNAs were selected to construct our prognostic model using LASSO Cox analysis, and 473 CRC patients were grouped according to risk scores. Then, we performed stratified sampling to check, validate and confirm our model by dividing 473 samples into a training group (*N* = 208) and a testing group (*N* = 205) in a 1:1 ratio. We reconfirmed the reliability and independence of the prognostic model through various methods and found the feasibility and practicality of applying the prognostic model to predict survival outcomes for different clinical features, such as age, lymph node status, and clinical stage. CRC patients with downregulated m6A-related gene expression, lower immune score, distant metastasis, lymph node metastasis, or advanced clinical staging had higher risk scores, indicating less-desirable outcomes. Furthermore, we explored the immune microenvironment (TME) of colorectal cancer cells.

There are still some limitations of this study. First, the data were obtained only from a single TCGA dataset. The analysis of multiple datasets would have been more convincing. Second, when analysing the immune microenvironment, we did not investigate the signalling pathways of the target genes at a deeper level. The immunological analysis of m6A-related prognostic lncRNAs and risk score led to a meaningful question: What is the specific mechanism of N6-methyladenosine-related lncRNAs and immune cells? This question deserves deeper research. There is still a long way to go to considerably optimise personalised immunotherapy management.

## Conclusion

In summary, we made an effort to illustrate the significance of m6A RNA modification regulators in colorectal cancer. The prognostic model we constructed may be used as an essential reference for predicting the outcome of CRC patients. With the increasing prevalence rate and early staging phenomenon, colorectal cancer patients urgently need precise personalised treatment.

This need undoubtedly places a higher demand on understanding the molecular science of colorectal cancer. Predicting the clinical outcome utilising a prognostic model will pave the way for targeted molecular treatment plans for colorectal cancer.

## Data Availability Statement

The original contributions presented in the study are included in the article/supplementary material, further inquiries can be directed to the corresponding authors.

## Ethics Statement

Written informed consent was obtained from the individual(s) for the publication of any potentially identifiable images or data included in this article.

## Author Contributions

EX, AB, HZ, and YX wrote the manuscript. YS and KR collected and analysed the raw data. LJ helped to revise the manuscript. AB designed the whole work. All authors contributed to the article and approved the submitted version.

## Conflict of Interest

The authors declare that the research was conducted in the absence of any commercial or financial relationships that could be construed as a potential conflict of interest.

## Publisher’s Note

All claims expressed in this article are solely those of the authors and do not necessarily represent those of their affiliated organizations, or those of the publisher, the editors and the reviewers. Any product that may be evaluated in this article, or claim that may be made by its manufacturer, is not guaranteed or endorsed by the publisher.

## References

[B1] Adams J. M. Cory S. (1975). Modified nucleosides and bizarre 5’-termini in mouse myeloma mRNA. *Nature* 255 28–33. 10.1038/255028a0 1128665

[B2] Bai Y. Yang C. Wu R. Huang L. Song S. Li W. (2019). Ythdf1 regulates tumorigenicity and cancer stem cell-like activity in human colorectal carcinoma. *Front. Oncol.* 9:332. 10.3389/fonc.2019.00332 31131257 PMC6509179

[B3] Beemon K. Keith J. (1977). Localization of N6-methyladenosine in the *Rous sarcoma* virus genome. *J. Mol. Biol.* 113 165–179. 10.1016/0022-2836(77)90047-x 196091

[B4] Chang R. Xiao X. Fu Y. Zhang C. Zhu X. Gao Y. (2021). Itgb1-Dt facilitates lung adenocarcinoma progression via forming a positive feedback loop with Itgb1/Wnt/β-Catenin/Myc. *Front. Cell Dev. Biol*. 9:631259. 10.3389/fcell.2021.631259 33763420 PMC7982827

[B5] Christensen L. L. True K. Hamilton M. P. Nielsen M. M. Damas N. D. Damgaard C. K. (2016). Snhg16 is regulated by the Wnt pathway in colorectal cancer and affects genes involved in lipid metabolism. *Mol. Oncol*. 10 1266–1282. 10.1016/j.molonc.2016.06.003 27396952 PMC5423192

[B6] Dekker E. Tanis P. J. Vleugels J. L. A. Kasi P. M. Wallace M. B. (2019). Colorectal cancer. *Lancet* 394 1467–1480. 31631858 10.1016/S0140-6736(19)32319-0

[B7] Deng R. Cheng Y. Ye S. Zhang J. Huang R. Li P. (2019). mA methyltransferase Mettl3 suppresses colorectal cancer proliferation and migration through p38/Erk pathways. *Onco Targets Ther.* 12 4391–4402. 10.2147/ott.s201052 31239708 PMC6556107

[B8] Ghasemi T. Khalaj-Kondori M. Hosseinpour Feizi M. A. Asadi P. (2020). lncRNA-miRNA-mRNA interaction network for colorectal cancer; an in silico analysis. *Comput. Biol. Chem.* 89:107370. 10.1016/j.compbiolchem.2020.107370 32932199

[B9] He L. Li J. Wang X. Ying Y. Xie H. Yan H. (2018). The dual role of N6-methyladenosine modification of RNAs is involved in human cancers. *J. Cell Mol. Med.* 22 4630–4639. 10.1111/jcmm.13804 30039919 PMC6156243

[B10] He X. Ma J. Zhang M. Cui J. Yang H. (2020). Long Non-Coding RNA Snhg16 activates Usp22 expression to promote colorectal cancer progression by sponging miR-132-3p. *Onco Targets Ther.* 13 4283–4294. 10.2147/ott.s244778 32547062 PMC7244243

[B11] Huang H. Weng H. Sun W. Qin X. Shi H. Wu H. (2018). Recognition of RNA N(6)-methyladenosine by Igf2bp proteins enhances mRNA stability and translation. *Nat. Cell Biol.* 20 285–295. 10.1038/s41556-018-0045-z 29476152 PMC5826585

[B12] Huang Q.-R. Pan X.-B. (2019). Prognostic lncRNAs, miRNAs, and mRNAs form a competing endogenous RNA network in colon cancer. *Front. Oncol.* 9:712. 10.3389/fonc.2019.00712 31448228 PMC6691151

[B13] Ke D. Wang Q. Ke S. Zou L. Wang Q. (2020). Long-Non Coding RNA Snhg16 supports colon cancer cell growth by modulating miR-302a-3p/Akt Axis. *Pathol. Oncol. Res.* 26 1605–1613. 10.1007/s12253-019-00743-9 31502038

[B14] Li T. Hu P. S. Zuo Z. Lin J. F. Li X. Wu Q. N. (2019). Mettl3 facilitates tumor progression via an m(6)A-Igf2bp2-dependent mechanism in colorectal carcinoma. *Mol. Cancer* 18:112. 10.1186/s12943-019-1038-7 PMC6589893 31230592

[B15] Li Y. Lu Y. Chen Y. (2019). Long non-coding RNA Snhg16 affects cell proliferation and predicts a poor prognosis in patients with colorectal cancer via sponging miR-200a-3p. *Biosci. Rep.* 39:BSR20182498. 10.1042/BSR20182498 PMC6522740 30962265

[B16] Liu H. Zhang Q. Lou Q. Zhang X. Cui Y. Wang P. (2020). Differential analysis of lncRNA, miRNA and mRNA expression profiles and the prognostic value of lncRNA in esophageal cancer. *Pathol. Oncol. Res.* 26 1029–1039. 10.1007/s12253-019-00655-8 30972633

[B17] Martinelli E. Ciardiello D. Martini G. Troiani T. Cardone C. Vitiello P. P. (2020). Implementing anti-epidermal growth factor receptor (Egfr) therapy in metastatic colorectal cancer: challenges and future perspectives. *Ann. Oncol.* 31 30–40. 10.1016/j.annonc.2019.10.007 31912793

[B18] Peng W. Li J. Chen R. Gu Q. Yang P. Qian W. (2019). Upregulated Mettl3 promotes metastasis of colorectal Cancer via miR-1246/Spred2/Mapk signaling pathway. *J. Exp. Clin. Cancer Res.* 38:393. 10.1186/s13046-019-1408-4 PMC6729001 31492150

[B19] Qi P. Xu M.-D. Ni S.-J. Shen X. H. Wei P. Huang D. (2015). Down-regulation of ncran, a long non-coding RNA, contributes to colorectal cancer cell migration and invasion and predicts poor overall survival for colorectal cancer patients. *Mol. Carcinog.* 54 742–750. 10.1002/mc.22137 24519959

[B20] Shen X. P. Ling X. Lu H. Zhou C. X. Zhang J. K. Yu Q. (2018). Low expression of microRNA-1266 promotes colorectal cancer progression via targeting Fto. *Eur. Rev. Med. pharmacol. Sci.* 22 8220–8226. 30556861 10.26355/eurrev_201812_16516

[B21] Sung H. Ferlay J. Siegel R. L. Laversanne M. Soerjomataram I. Jemal A. (2021). Global cancer statistics 2020: GLOBOCAN estimates of incidence and mortality worldwide for 36 cancers in 185 countries. *CA Cancer J. Clin.* 71 209–249. 10.3322/caac.21660 33538338

[B22] Taieb J. Le Malicot K. Shi Q. Penault-Llorca F. Bouché O. Tabernero J. (2017). Prognostic value of braf and kras mutations in MSI and MSS stage III colon cancer. *J. Natl. Cancer Inst.* 109:djw272. 10.1093/jnci/djw272 28040692 PMC6075212

[B23] Tanabe A. Tanikawa K. Tsunetomi M. Takai K. Ikeda H. Konno J. (2016). RNA helicase Ythdc2 promotes cancer metastasis via the enhancement of the efficiency by which Hif-1α mRNA is translated. *Cancer Lett.* 376 34–42. 10.1016/j.canlet.2016.02.022 26996300

[B24] Wang Y. Lu J. H. Wu Q. N. Jin Y. Wang D. S. Chen Y. X. (2019). LncRNA Linris stabilizes Igf2bp2 and promotes the aerobic glycolysis in colorectal cancer. *Mol. Cancer* 18:174. 10.1186/s12943-019-1105-0 PMC6886219 31791342

[B25] Wu Y. Yang X. Chen Z. Tian L. Jiang G. Chen F. (2019). m(6)A-induced lncRNA Rp11 triggers the dissemination of colorectal cancer cells via upregulation of Zeb1. *Mol. Cancer* 18:87. 10.1186/s12943-019-1014-2 PMC6461827 30979372

[B26] Xie Y. Gu J. Qin Z. Ren Z. Wang Y. Shi H. (2020). Long non-coding RNA Fam66C is associated with clinical progression and promotes cell proliferation by inhibiting proteasome pathway in prostate cancer. *Cell Biochem. Funct.* 38 1006–1016. 10.1002/cbf.3531 32430927

[B27] Zhang C. Dang D. Liu C. Wang Y. Cong X. (2021). Identification of tumor mutation burden-related hub genes and the underlying mechanism in melanoma. *J. Cancer* 12 2440–2449. 10.7150/jca.53697 33758620 PMC7974884

[B28] Zhou J. Zheng R. Zhang S. Zeng H. Wang S. Chen R. (2021). Colorectal cancer burden and trends: comparison between China and major burden countries in the world. *Chin. J. Cancer Res.* 33 1–10. 10.21147/j.issn.1000-9604.2021.01.01 33707923 PMC7941684

[B29] Zhou Z. Lv J. Yu H. Han J. Yang X. Feng D. (2020). Mechanism of RNA modification N6-methyladenosine in human cancer. *Mol. Cancer* 19:104. 10.1186/s12943-020-01216-3 PMC7278081 32513173

